# Applicability of Confocal Raman Microscopy to Observe Microstructural Modifications of Cream Cheeses as Influenced by Freezing

**DOI:** 10.3390/foods9050679

**Published:** 2020-05-25

**Authors:** Marcello Alinovi, Germano Mucchetti, Ulf Andersen, Tijs A. M. Rovers, Betina Mikkelsen, Lars Wiking, Milena Corredig

**Affiliations:** 1 Food and Drug, University of Parma, Parco Area delle Scienze, 47/A 43124 Parma, Italy; germano.mucchetti@unipr.it; 2 Arla Innovation Centre, Arla Foods, Agro Food Park 19, 8200 Aarhus, Denmark; ulf.andersen@arlafoods.com (U.A.); tijs.albert.maria.rovers@arlafoods.com (T.A.M.R.); bm@arlafoods.com (B.M.); 3 Department of Food Science and iFOOD Center for Innovative Food, Aarhus University, Agro Food Park 48, 8200 Aarhus, Denmark; lars.wiking@food.au.dk (L.W.); mc@food.au.dk (M.C.)

**Keywords:** microstructure, Raman spectroscopy, confocal laser scanning microscopy, cheese freezing, cream cheese, NMR spectroscopy, cryoprotectants

## Abstract

Confocal Raman microscopy is a promising technique to derive information about microstructure, with minimal sample disruption. Raman emission bands are highly specific to molecular structure and with Raman spectroscopy it is thus possible to observe different classes of molecules in situ, in complex food matrices, without employing fluorescent dyes. In this work confocal Raman microscopy was employed to observe microstructural changes occurring after freezing and thawing in high-moisture cheeses, and the observations were compared to those obtained with confocal laser scanning microscopy. Two commercially available cream cheese products were imaged with both microscopy techniques. The lower resolution (1 µm/pixel) of confocal Raman microscopy prevented the observation of particles smaller than 1 µm that may be part of the structure (e.g., sugars). With confocal Raman microscopy it was possible to identify and map the large water domains formed during freezing and thawing in high-moisture cream cheese. The results were supported also by low resolution NMR analysis. NMR and Raman microscopy are complementary techniques that can be employed to distinguish between the two different commercial formulations, and different destabilization levels.

## 1. Introduction

When a sample is subjected to a monochromatic light source, a small proportion of the radiation is scattered depending on the physical and chemical properties of its components. The interactions between the laser light and the molecular vibrations cause a shift in energy between the incident and the scattered light. Vibrational spectroscopy methods such as infrared, mid- and near-infrared, and Raman are commonly used to assess structures and identify molecular species. Raman scattering spectra are specific to the chemical functional groups and their bonds’ vibrational frequency, and can be used to distinguish molecular structures [1]. In Raman microscopy, the emission spectra are collected at high spatial resolution, and the band pattern is specific to the sample’s composition. Furthermore the intensity is related to the concentration of specific component, if the signal of such component is sufficient to overcome the signal-to-noise ratio. Raman microscopy has gained interest as a tool to characterize food products in situ, with minimal sample preparation. This technique has been reported to identify components (fat, proteins, water) in polyphasic systems such as mayonnaise, semi-hard Swiss cheese, and soymilk [2]. Raman microscopy has been employed to investigate differences in composition of milk fat globules [3], to classify cheeses on the basis of their different microstructural organization, to process spectra with the aid of multivariate tools [4], to study the formation and the cause of structural modifications (i.e., crystallization) during ripening in hard and extra-hard cheeses [5]. Furthermore, the microstructural features of a cheese matrix have been analyzed by Raman microscopy, by observing the spatial distribution of ingredients (macromolecules, water, paprika, trisodium citrate, phospholipids) in Cheddar and imitation cheeses [6,7]. Both qualitative and quantitative analysis can be carried out by Raman microscopy. Because of the distinct signal of different chemical species, the use of fluorescence dyes in sample preparation is not necessary. It has been recently shown that it is possible to resolve spatially the NaCl concentration in butter samples [8] and to predict the solid fat content of anhydrous milk fat [1]. In sum, confocal Raman microscopy may be applied not only to observe food microstructure but also to gain information about geometries, distances, angles, and polarizability of the chemical bonds present in the structure for the different components of the matrix [1,9].

These characteristics may represent an important advantage of confocal Raman microscopy if compared to the more conventional non-disruptive technique used to study the microstructure of complex food matrices [10]: confocal laser scanning microscopy. Confocal laser scanning microscopy necessitates specific dyes to observe different compounds and therefore it requires some sample preparation. Furthermore, the observations would be limited by the dies and their interactions with the molecules, and the number of wavelengths (i.e., laser sources) available for the observation [11].

In this work, confocal Raman microscopy was applied together with laser scanning microscopy to investigate the microstructure of high moisture cheese, and to identify changes in the distribution of the molecular species caused by processing and storage; the comparison of the obtained results can be useful to benchmark and to compare the benefits and disadvantages related to both microscopy techniques. Despite the high potential of Raman microscopy, this technique has some drawbacks: it can be time-consuming, and the laser beam applied in Raman spectroscopy can heat, dry or damage the sample [12]. Long analysis times and the increase in temperature of the sample can result in analytical challenges (e.g., moisture losses, free surface flows, thickness variations), especially in high moisture samples. Furthermore, in spite of the chemical specificity, some of the components may not be detected in mixed systems, because of low signal thresholds [13,14].

Cream cheese is a soft, fresh cheese with a slightly acidic taste [15,16]. This cheese variety is characterized by a moisture content usually higher than 65%. Cream cheeses can also be categorized according to their fat content [16]. To obtain the right structure, the fat to protein ratio with the high moisture content are often balanced by the application of heat treatments of the cheese, in order to increase the interactions between whey proteins, caseins and water [16]. As for yogurt products, to encounter the preference of a larger number of consumers the acidic taste of cream cheese may be modified by addition of sugars.

Depending on commercial standards, this product may contain various stabilizers and emulsifiers that modify its structure and functionality, properties that are especially important when cream cheese is used for further processing. Freezing is an important process applied to cream cheese in cases where robust supply chains are needed, for example, to extend market reach, to provide food service customers with a consistent product, or to decrease waste and improve shelf life by reducing the rate of degradation during refrigerated storage [17,18,19]. Hence, freezing is becoming a convenient tool for export or when the product is used as an ingredient for further manufacturing [20]. However, from a physical point of view, freezing can have a negative impact on the cheese matrix: it can cause the rupture of the casein matrix as a consequence of ice crystals formation, with the creation of voids and large serum channels in the structure [21,22], the modification of the water status and distribution (e.g., protein dehydration phenomena) [23], and some rheological and sensory changes [17].

In cream cheese products, non-dairy ingredients are often added to improve technological functionalities. To preserve the structure of the protein network, which partially gives the cheese its characteristics, and to limit freezing-destabilization, different additives are used. Sorbitol, polyphosphate, gelatin, polyols, sucrose and other sugars or polysaccharides are often incorporated as cryoprotectants [24] and act via different mechanisms. Sugars and polyols stabilize proteins through their effect on the water fraction and modification of solvent quality, critical to keep the protein network intact. Carbohydrates or polyols can also cause the formation of hydrogen bonds with protein side chains and increase protein hydration while decreasing protein-protein interactions. Polysaccharides can bind water and form large complexes, and increase the viscosity of the continuous phase.

This project focuses on evaluating the potential application of confocal Raman microstructure tools to observe changes in the structure and in the distribution of the components. In particular, to evaluate the ability to observe changes in the quality of cream cheese after freezing and thawing with confocal Raman microscopy, compared to confocal laser scanning microscopy. The observations are supported by measurements of water mobility using low resolution NMR, for products subjected to freezing and thawing. Two commercial cream cheeses with a different protein to fat ratio and containing different amounts and types of stabilizers, and expected to have different stabilities during freezing and thawing, were used as model systems in this study.

## 2. Materials and Methods

### 2.1. Cream Cheese Treatments

Two different commercial full fat cream cheese products (A and B) were selected as having a different composition in terms of macro components and stabilizers, their composition is reported in Table 1. In particular, cream cheese B had a higher protein and carbohydrates content, and a higher number of stabilizers in its formulation, than cream cheese A. The cheese samples were collected and processed in portions of 180 g and stored in polypropylene plastic boxes (2.5 cm × 10 cm × 7 cm). Samples were frozen at −20 °C in still air conditions; after 7 days of frozen storage at −20 °C, cheeses were thawed at 4 °C overnight. Analyses were conducted on non-frozen cheese before freezing (control sample), and after thawing. For each treatment, at least 5 replicates were performed, or 5 images were obtained for each measured sample.

### 2.2. Cheese Preparation for Microscopy

Cream cheese samples stored at 4 °C were sectioned with a knife in a thin layer of approximately 5 mm and were then positioned on a microscope slide. Another microscope slide was placed on the upper layer and sample’s outer layer was coated with paraffin oil to avoid moisture evaporation during analysis. Samples were left to equilibrate at 22 °C for 15 min in order to reduce possible changes of thickness caused to by temperature changes during the analysis. For laser scanning confocal measurements, Nile red and fluorescein isothiocyanate (FITC) were used as fluorescent staining agents for fats and proteins, respectively. Both staining agents were dissolved in acetone (0.01%). One or two drops of staining solution were placed on a microscope slide and acetone was allowed to evaporate before sample addition, as previously described [25]. Samples were left in contact with the dyes for approximately 5 min before imaging.

### 2.3. Microstructural Analyses

Microstructural analyses of the cheeses were carried out using a Nikon Eclipse Ti2 confocal laser scanning microscope (Minato, Tokyo, Japan) equipped with 10×, 40× and 100× objectives. The Ar/Kr and HeNe laser beam were set at 488 nm and 561 nm, respectively. The micrographs were acquired using the 40× objective (with the exception of Figures 6 and 7 that were acquired with the 10× objective) in 1024 × 1024 pixels sections, with each image covering an area of 375 × 375 μm; each image was obtained as the average of four frames. As the laser penetration depth was lower than 10–15 µm according to Auty [26], samples were imaged close to the surface of each specimen.

Raman images obtained using an Alpha 300 R instrument (WITec, Ulm, Germany) were produced by taking 50 × 50 Raman spectra in a uniform 50 × 50 µm grid, with a final resolution of 1 μm/px. A laser wavelength of 532 nm with a laser power of 20 mW was used. Laser intensity value was chosen as it was the highest one that did not cause an excessive temperature increase of the sample, that could possibly alter the cheese characteristics during the analysis. Despite the maximum depth penetration of the laser source was higher than 40–50 μm, the measurements were performed at a penetration depth of ~10 μm, in order to maximize the signal intensity [27,28]. A 50× objective was used to observe microstructure of samples; an integration time of 0.20–0.10 s was used, depending on sample characteristics and stability. Analysis time was about 5–10 min depending on the integration time used. Data were computed into microstructure images using WITec Suite FIVE software package. Cosmic ray removal, Savitzky Golay smoothing and baseline offset correction were used as pre-processing methods to improve spectra quality and to reduce noise, without the loss of any important information [6]. Microstructural images were created from the hyperspectral datasets by performing a multivariate clustering analysis and the identification of the components was made by comparing spectral data of the samples with a reference database of cheese ingredients. Reference spectra were collected for all the ingredients present in the cheese formulations, as indicated in Table S1 (Supplementary Material). Raman spectra were collected using a 10× objective, a laser power of 30 mW, an integration time of 5 s and performing 30 accumulations. Reference spectra were finally obtained by performing three single acquisitions in different sampling points of the surface of the reference sample and by averaging the signals.

### 2.4. NMR Analysis

Proton self-diffusion coefficient (D) measurements were carried out using a low-field NMR spectrometer (Bruker MiniSpec, Bruker BioSpin GmbH, Rheinstetten, Germany) operating at 20 MHz and at a temperature of 20 °C with a Pulsed Field Gradient Spin-Echo (PFG-SE) method. The probe was calibrated with a solution containing 1.25 g/L CuSO_4_ 5H_2_O and pure water (D = 1.98 × 10^−9^ m^2^ s^−1^ at 20 °C) at 10, 30, 50% pulsed gradient amplitude. For each measurement, a total of 16 echoes were acquired as a function of the gradient pulse duration using a recycle delay of 2 s. The gradient pulse width was set at 0.5 ms, and the gradient pulse separation (Δ) was set to 7.5 ms. Approximately 4.5 g of samples were placed into NMR tubes (10 mm external diameter). The tubes were filled with sample to about 15 mm height. A gradient pulse of 40% was used for the analyses.

### 2.5. Statistical Analysis

^1^H self-diffusion coefficient (D) data were statistically analyzed for both the cream cheese formulations to observe possible effects related to the freezing and thawing processes. One-way Analysis of Variance (ANOVA) was carried out using SPSS v.25 (IBM, Armonk, NY, USA), considering a significance level α = 0.05.

## 3. Results

### 3.1. Raman Spectra Database Collection

**Figure 1:**
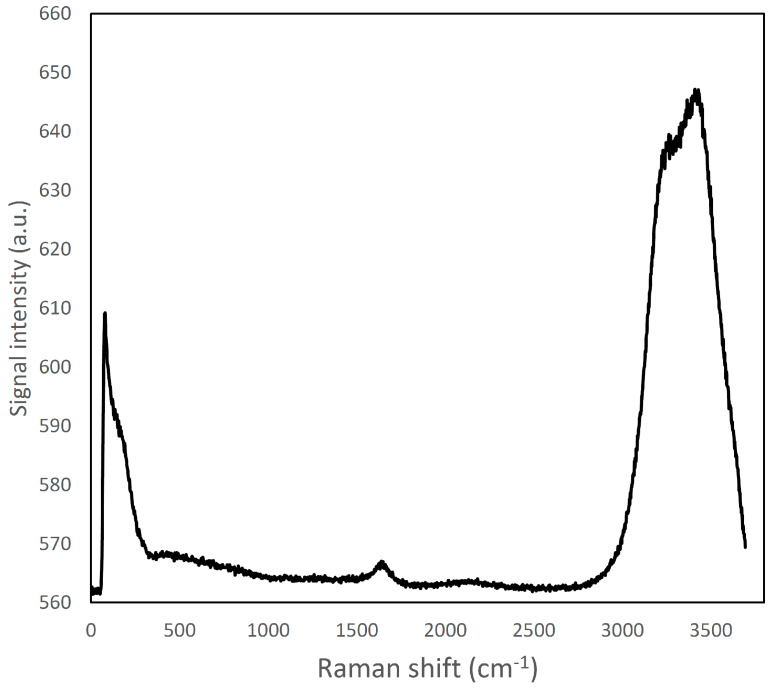
Raman spectrum of ultra-pure water; a large emission band can be viewed in the region around 2900–3800 cm^−1^.

**Figure 2:**
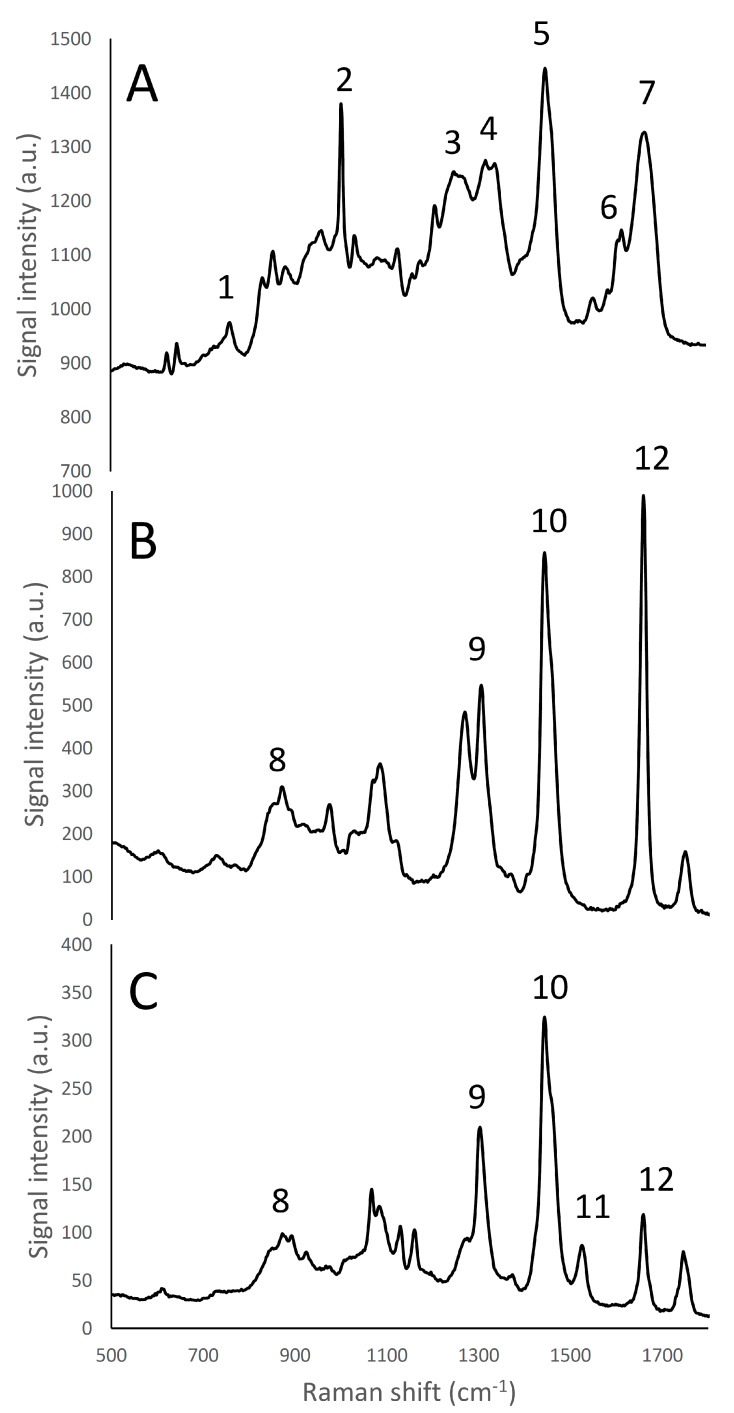
Raman fingerprint region (500–1800 cm^−1^) of micellar casein isolate (**A**), rapeseed oil (**B**), and butter (**C**). Panel A: (1) tryptophan indole ring (750–760 cm^−1^); (2) phenylalanine benzene ring breathing (1000–1005 cm^−1^); (3) amide III β-sheet structure (1270 cm^−1^); (4) amide III α -helices structure (1320–1340 cm^−1^) (5) CH_2_ bending peak (1450 cm^−1^); (6) tyrosine (1614 cm^−1^); (7) amide I (1650–1670 cm^−1^). Panels B, C: (8) phospholipids headgroup (870 cm^−1^); (9) CH_2_ twisting of phospholipids (1300–1310 cm^−1^); (10) CH_2_ scissoring of sterols (1442 cm^−1^); (11) Carotenoids stretching band (1525 cm^−1^); (12) C=C *cis* stretching of phospholipids (1655 cm^−1^).

Reference spectra were collected for the ingredients present in the cheese samples. The whole spectra dataset is reported as Supplemental Information (Figures S1–S14, Supplementary Material). Figure 1 and Figure 2 show the spectra collected for the main components present in the cream cheese samples: water, casein proteins (measured as micellar casein powder), and the lipid fraction, that was constituted by both butter and rapeseed oil. Carrageenan, citrus fibre, gelatin and locust bean gum did not show Raman emission peaks when measured in reference samples. For these components, it was just possible to observe a variation of the baseline at different wavenumbers, as already reported by Yang et al. [13]. On the other hand, it was possible to identify peaks of sorbic acid molecules (Figure S2, Supplementary Material), a strong peak was observable at 1635 cm^−1^ which was attributed to C=O or C-C stretches; identification was made according to Kai et al. [29].

Sugars (lactose as a residue of fermentation and sucrose, mainly in cheese B) that were present in both formulations, showed several emission peaks in the range between 300 and 1700 cm^−1^ (Figures S8 and S9, Supplementary Material).

**Figure 3:**
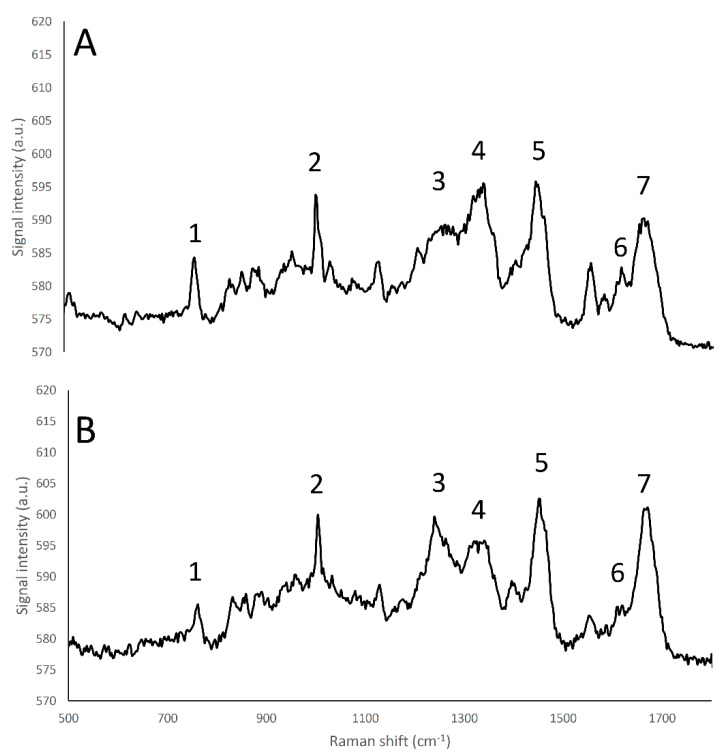
Raman fingerprint region (500–1800 cm^−1^) of α-lactalbumin (**A**), and β-lactoglobulin (**B**). (1) tryptophan indole ring (750–760 cm^−1^); (2) Phenylalanine benzene ring breathing (1000–1005 cm^−1^); (3) amide III β-sheet structure (1270 cm^−1^); (4) amide α -helices structure (1320–1340 cm^−1^); (5) CH_2_ bend peak (1450 cm^−1^); (6) tyrosine (1614 cm^−1^); (7) amide I (1650–1670 cm^−1^).

The Raman emission spectrum of water showed a strong, broad peak around 2900–3800 cm^−1^ (Figure 1), far away from the fingerprint region of many molecules, including fat and protein [6]. Fat and protein components showed a large peak around 2800–3000 cm^−1^ that can be attributed to CH_2_, CH_3_ stretching; however, because of this overlapping, this signal cannot be used to identify and to discriminate the single components. The most important region of the spectra is located in the range between 500–1800 cm^−1^. This spectral region contains fingerprint peaks that are typical of a class of components (i.e., fat or protein) or of single molecules [2] and that can be useful to identify and map the presence and the distribution of the different components in a complex matrix. In particular, proteins are characterized by 7 important peaks: tryptophan indole ring (750–760 cm^−1^), phenylalanine ring breathing (1000–1005 cm^−1^), β-sheets and α-helices structures related to amide III (1270 cm^−1^ and 1320–1340 cm^−1^, respectively), CH_2_ bending peak (1450 cm^−1^), tyrosine (1614 cm^−1^), and Amide I (1650–1670 cm^−1^), and, as it can be observed in details in Figures S13 and S14 (Supplementary Material) and in Figure 2A and Figure 3 [6,30,31,32].

Of these Raman emission peaks, the phenylalanine peak has been proved to be the most useful to visualize the protein distribution in cheeses, because of the absence of other interfering emission peaks in its spectral range [6].

The whey proteins α-lactalbumin and β-lactoglobulin showed different relative amide III peak intensities that are related to differences in terms of secondary protein structure, as the α-lactalbumin structure is mainly composed by α-helices, and β-lactoglobulin is mainly characterized by β-sheets [33]; in particular, the band located at 1240 cm^−1^, corresponding to β-sheet secondary structures of amide III [32] had an higher intensity in β-lactoglobulin than in α -lactalbumin spectrum, while conversely the band located at 1320–1340 cm^−1^, corresponding to α-helix secondary structures of amide III [32], showed a higher intensity in the case of α -lactalbumin than β-lactoglobulin (Figure 3).

The lipid fraction showed an important peak, representing the CH_2_ scissoring of sterols at 1442 cm^−1^ (Figure 2B,C). Other peaks that could be identified [6] corresponded to: the CH_2_ twisting of phospholipids (1300–1310 cm^−1^), phospholipids headgroup (870 cm^−1^) and C=C cis stretching of phospholipids (1655 cm^−1^). Furthermore, rapeseed oil and butter, containing different quantities of saturated and unsaturated fatty acids, show different intensities of the peak at 1655 cm^−1^. Butter is also characterized by the presence of a carotenoid stretching band at 1525 cm^−1^. These peaks could also be used to quantify or identify and discriminate the presence of different lipid components in products with unknown composition, by the development of proper calibrations [34]. From Figure 2B,C it was possible to note one of the most intense fat emission peak was the CH_2_ scissoring of sterols, in accordance with Gallier et al. [3]. This peak has already been used to map with Raman microscopy the fat distribution in cheese matrices [6].

In the following Raman microscopy observations of cream cheese, the proteins were identified using the phenylalanine’s benzene ring breathing peak (1000–1005 cm^−1^) or the amide III peak related to the β-sheets structure (1270 cm^−1^), while the lipid molecules were visualized by the CH_2_ scissoring band of sterols (1442 cm^−1^).

### 3.2. Microstructural Observations

**Figure 4:**
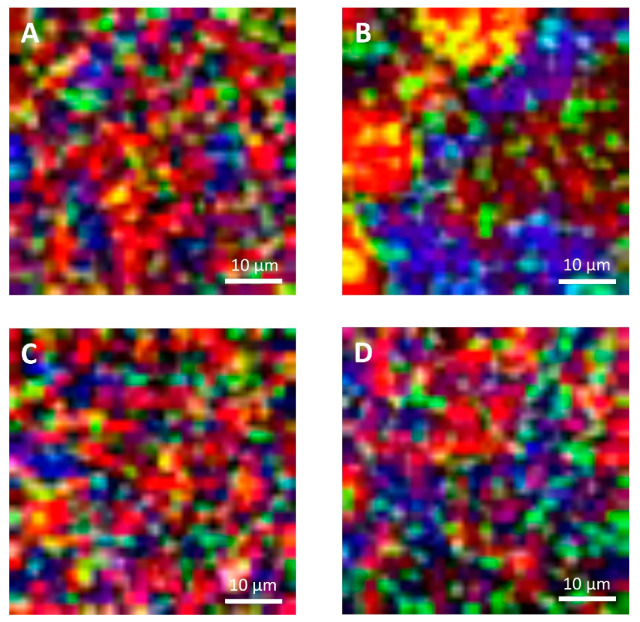
Confocal Raman micrographs of cream cheeses. Cream cheese A before freezing (**A**), after freezing (**B**). Cream cheese B before freezing (**C**), after freezing (**D**). Red: fat. Green: protein. Blue: water.

**Figure 5:**
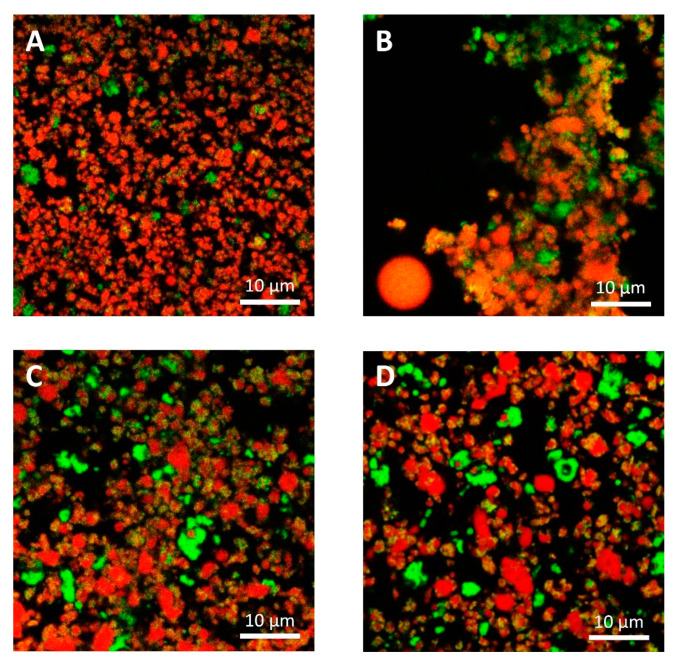
Confocal laser scanning micrographs of cream cheeses. Cream cheese A before freezing (**A**), after freezing (**B**). Cream cheese B before freezing (**C**), after freezing (**D**). Red: fat. Green: protein.

The microstructural observations of the cheese samples obtained by confocal Raman microscopy (Figure 4) were in agreement with those obtained with conventional laser scanning microscopy (Figure 5) in terms of microstructural changes caused by freezing and thawing for the protein and fat domain. Moreover, these observations were representative of the cheese samples, as observable from microstructure observations made on different sampling aliquots (Figures S15 and S16). In the case of confocal Raman microscopy, it was possible to obtain additional information regarding the distribution of water in the samples, thanks to the peak outside the fingerprint region (3000–3800 cm^−1^). This information was not obtainable with confocal laser scanning microscopy: in this case the appearance of voids in between the protein and fat domains may correspond to the serum domain and other water-soluble compounds present in the cheeses.

On the contrary, because of the limitations imposed to acquisition parameters that were selected to guarantee the samples’ stability during analyses and also to limit the time required for each measurement, Raman micrographs were characterized by a limited and lower image resolution than conventional laser scanning microscopy. Because of this limitation, with Raman microscopy it was not possible observe fat globules, protein aggregates or other components having dimensions lower than 1 µm, that were observable with confocal laser scanning microscopy (Figure 5). Moreover, according to this limitation it was also not possible to detect carbohydrates (mainly sugars) that were present in high concentrations in both formulations, and some of the additives present in low concentrations. For example, it was not possible to detect sucrose, lactose, and sorbic acid, with the last one that showed a strong intensity of its signal with a peak at 1629 cm^−1^ in the reference sample (Figure S2, Supplementary Material).

With confocal laser scanning microscopy, fresh cream cheeses A and B showed a slightly different microstructural organization: cheese B compared to cheese A showed the presence of slightly bigger fat globules and fat globule aggregates that form clusters (Figure 5A,C); moreover, the lipid fraction of cheese B was surrounded by a denser protein matrix, compared to cheese A, in accordance with their higher protein content (Table 1). These differences were not visible in Raman observations (Figure 4A,C), as a consequence of the limited resolution of the acquired images.

With both microscopy techniques, it was possible to observe that in both formulations, a proteins fraction was adsorbed on the surface of fat globules. Cheese B showed a higher amount of adsorbed protein probably because the higher protein/fat ratio (0.36, if compared to 0.23 of cheese A) [35].

In Figure 3A,C and Figure 4A,C, it was also possible to visualize voids around fat globules and the protein matrix. These voids probably contained other ingredients (stabilizers, sugars, etc.) dispersed in the interstitial solution. These components were not directly detected neither by the confocal Raman or the conventional laser scanning microscopy procedures. The differences in the protein distribution at the interface, and the organization of the fat globules clusters, may have a role in modulating the freeze-thawing stability of the different cheese formulations; for example, a higher amount of adsorbed proteins on the surface of fat globules/clusters may result in a reduction of fat globules clustering and coalescence, and an improved stability.

**Figure 6:**
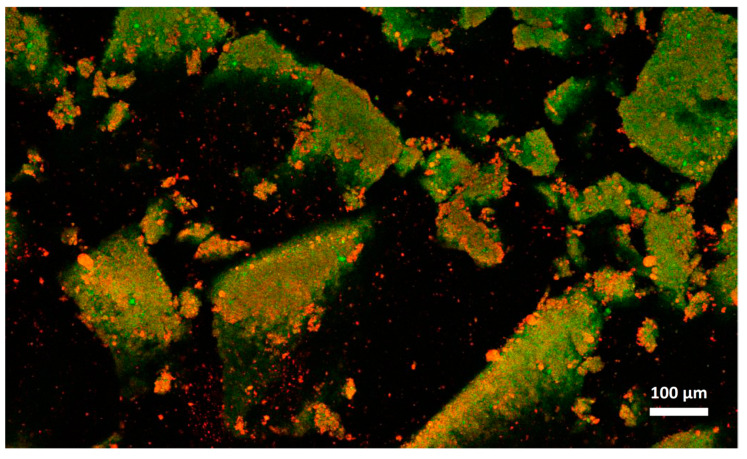
Microstructure of cream cheese A observed with confocal laser scanning microscope after freezing and thawing. Red: fat. Green: protein.

**Figure 7:**
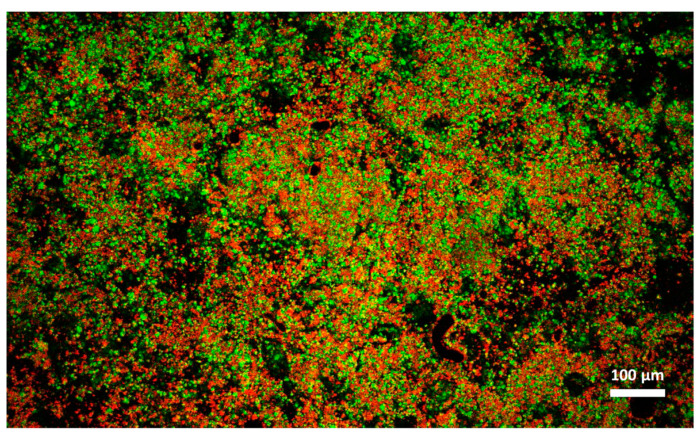
Microstructure of cream cheese B observed with confocal laser scanning microscope after freezing and thawing. Red: fat. Green: protein.

Cheese A showed significant destabilization after thawing, fat aggregation and the formation of clumps/granules of proteins/fat (Figure 6) with large channels of free water (Figure 4B). Thus, it was clear that in this sample, a strong separation of water from the solid components of the cheese occurred after freezing. On the other hand, cream cheese B appeared less modified by freezing (Figure 7) and its microstructure was substantially different from that of frozen thawed cheese A (Figure 6). There were smaller changes in this sample: it was still possible to observe slightly larger voids between the protein/fat structure in the frozen-thawed sample (Figure 4 and Figure 5D) compared to the unfrozen control (Figure 4 and Figure 5C), and this microstructure difference was attributed to a slight separation of water and the formation of larger serum channels (Figure 4D).

The two model systems were chosen as differences in formulations would result in differences in freezing stability: cheese B, contained more stabilizers, proteins and a higher carbohydrates and sugar content (Table 1) than cheese A. As expected, Cheese B was more stable, and showed only slight structural modifications, because of the effect of these ingredients acting as cryoprotectants and in general as stabilizers during freezing and thawing.

### 3.3. Water Mobility Changes in Cream Cheese after Thawing

The microstructure observations described in the previous section (3.2) were supported by low resolution NMR measurements on the two cheese samples. As reported in Table 2, an increase in the translational mobility of the water protons, was reflected by the increased D values after freezing. The ^1^H self-diffusion coefficient (D) measured by low resolution NMR showed a significant increase (*p* < 0.05) for cheese B, from 0.624 × 10^−9^ m^2^ s^−1^ of the control, unfrozen cheese, to 0.651 × 10^−9^ m^2^ s^−1^ after freezing and thawing. However, in the case of cheese A, the diffusion coefficient did not show a significant change (*p* > 0.05). This was due to the large variability of the matrix after freezing and thawing, resulting in a high standard deviation of the ^1^H self-diffusion coefficient (30% coefficient of variation). It is well known that freezing process can damage the structure of cheese [36,37,38]. In particular, it has been reported that casein gels may rearrange due to the growth of ice crystals; as water migrates to form ice crystals, protein-protein interactions occur [39]. After thawing, the protein structures may not be able to rebind the amount of water depleted, resulting in a higher value of D and a larger amount of unbound water. Hori [20] reported that the amount of unbound water in frozen-thawed cream cheeses measured by NMR is inversely proportional to the rates of freezing-thawing processes, and directly proportional to the extent of freeze and thaw-induced damages.

In cream cheese A, the freezing-thawing treatments caused a high degree of destabilization and non-homogeneity in the physical structure of the cheese, as already pointed out by the high standard deviation of D coefficient; this phenomenon was also highlighted by Raman and laser scanning microscopical observations that showed the presence of solid clumps of proteins-fat surrounded by channels of free water; conversely, this was not observed in the case of cheese B (Figure 4, Figure 5, Figure 6 and Figure 7). While the lack of homogeneity was clearly shown in microstructure measurements, NMR analyses underestimated the D values in the solid portion of the cheese, and a higher D value in the areas rich in free serum, in non-homogenous samples.

## 4. Conclusions

Confocal Raman microscopy showed the potential to be applied to study process-related microstructural modifications in high moisture spreadable dairy products, such as cream cheese. With Raman microscopy it was possible to observe structural differences, especially in terms of phase separation and water pockets re-organization. On the other hand, under the conditions reported in this work, with confocal laser scanning microscopy better results were obtained, in terms of fat and protein structure visualization. The use of confocal Raman microscopy allows one to better distinguish the effect of freezing-thawing processes of the two different cream cheeses. This was coherent with the low resolution of Raman microscopy obtained with the measurement parameters applied in this study; the parameters were limited in order to avoid any possible sample damage or modification that could be expected by a higher extent of heat generated by the laser source in the case of a slower but more resolute scan. Therefore, it was not possible to visualize the presence and distribution of sugars and different stabilizers, which probably contributed to the different freezing stability of the cheeses. It is also important to note that the Raman microscopy data complemented the information derived from low resolution NMR.

These first results suggest that confocal Raman microscopy may become in the future an additional key analytical tool because it can provide relatively rapid, non-destructive and online food quality evaluation, that could be complementary to confocal laser scanning microscopy.

Further studies need to be carried out on the quantitative assessment of components and ingredients, by image analysis techniques and/or by further elaborating spectral information.

## Figures and Tables

**Table 1:** Compositional information of full fat cream cheese samples.

Composition (g/100 g)	Cream Cheese A	Cream Cheese B
Fat	23.0	24.0
Carbohydrates	3.4	15.0
Fibre	1.1	1.5
Protein	5.4	8.7
Salt	0.72	0.58
Stabilizers	Carrageenan, locust bean gum	Carrageenan, locust bean gum, gelatin, citrus fibre

**Table 2:** Averaged ^1^H self-diffusion coefficient (D) (× 10^−9^ m^2^ s^−1^) of cream cheese formulations A and B before and after freezing and thawing.

Treatment	Cheese A (*n* = 5)		Cheese B (*n* = 5)	
	Mean D (×10^−9^ m^2^ s^−1^)	Standard Deviation	Mean D (×10^−9^ m^2^ s^−1^)	Standard Deviation
Control cheese	0.927	0.005	0.624 *	0.007
Frozen-thawed cheese	1.05	0.31	0.651 *	0.019

* Asterisks within the same column indicate significant difference (*p* < 0.05).

## Supplementary Materials

The following are available online at https://www.mdpi.com/2304-8158/9/5/679/s1, Figure S1–S12: Raman spectra of reference samples: (1) Carrageenan, (2) Sorbic acid, (3) Citrus fibre, (4) Gelatine, (5) α-lactalbumin, (6) β-lactoglobulin (7) Whey protein concentrate, (8) Sucrose, (9) Lactose, (10) Skim milk powder, (11) Micellar casein isolate, (12) Calcium caseinate, Figure S13–S14: Raman fingerprint region (500-1800 cm^-1^) of protein components: (13) Calcium caseinate, (14) Whey protein concentrate. (A) Phenylalanine’s benzene ring breathing (1000-5 cm^-1^); (B) Amide III (1270 cm^-1^); (C) Tyrosine (1614 cm^-1^); (D) Amide I (1650-70 cm^-1^), Figure S15: Supplementary Confocal Raman micrographs of cream cheeses. Cream cheese A before freezing (A), after freezing (B). Cream cheese B before freezing (C), after freezing (D). Red: fat. Green: protein. Blue: Water, Figure S16. Supplementary Confocal laser scanning micrographs of cream cheeses. Cream cheese A before freezing (A), after freezing (B). Cream cheese B before freezing (C), after freezing (D). Red: fat. Green: protein. Table S1: Reference samples used to acquire Raman spectroscopy peaks.
