# Supplementary material for: Applicability of Confocal Raman Microscopy to Observe Microstructural Modifications of Cream Cheeses as Influenced by Freezing
Source: Foods. 2020 May 25;9(5):679. doi: 10.3390/foods9050679 (PMC7278691; doi:10.3390/foods9050679)

SUPPLEMENTARY MATERIAL

Table S1. Reference samples used to acquire Raman spectroscopy peaks.

Sample	Company/Producer	Component/Ingredient	State
MilliQ Water	Arla	Water	liquid
Clarified butter	Arla	Fat	solid
Rapeseed oil	Arla	Fat	liquid
Micellar casein isolate	Arla	Caseins	powder
Calcium Caseinate	Arla	Caseins	powder
Whey protein concentrate	Arla	Whey proteins	powder
Alpha lactalbumin	Sigma Aldrich	-	powder
Beta lactoglobulin	Sigma Aldrich	-	powder
Lactose	Arla	-	powder
Sucrose	Sigma-Aldrich	-	powder
Carrageenan	Arla	-	powder
Locust bean gum	Arla	Locust bean gum, sorbic acid	powder
Citrus fibre	Arla	-	powder
Gelatine	Arla	-	powder
Skimmed milk powder	Arla	Proteins, lactose	powder

Figure S1-12. Raman spectra of reference samples: 1) Carrageenan, 2) Sorbic acid, 3) Citrus fibre, 4) Gelatine, 5) α-lactalbumin, 6) β-lactoglobulin 7) Whey protein concentrate, 8) Sucrose, 9) Lactose, 10) Skim milk powder, 11) Micellar casein isolate, 12) Calcium caseinate.

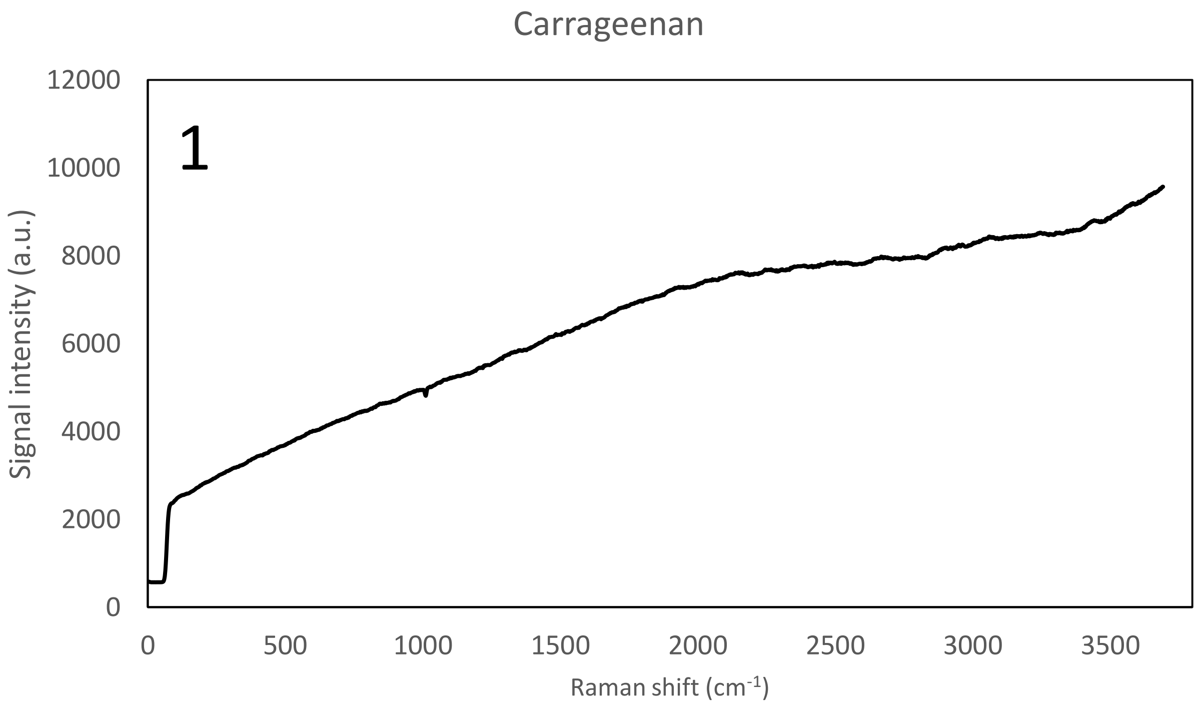

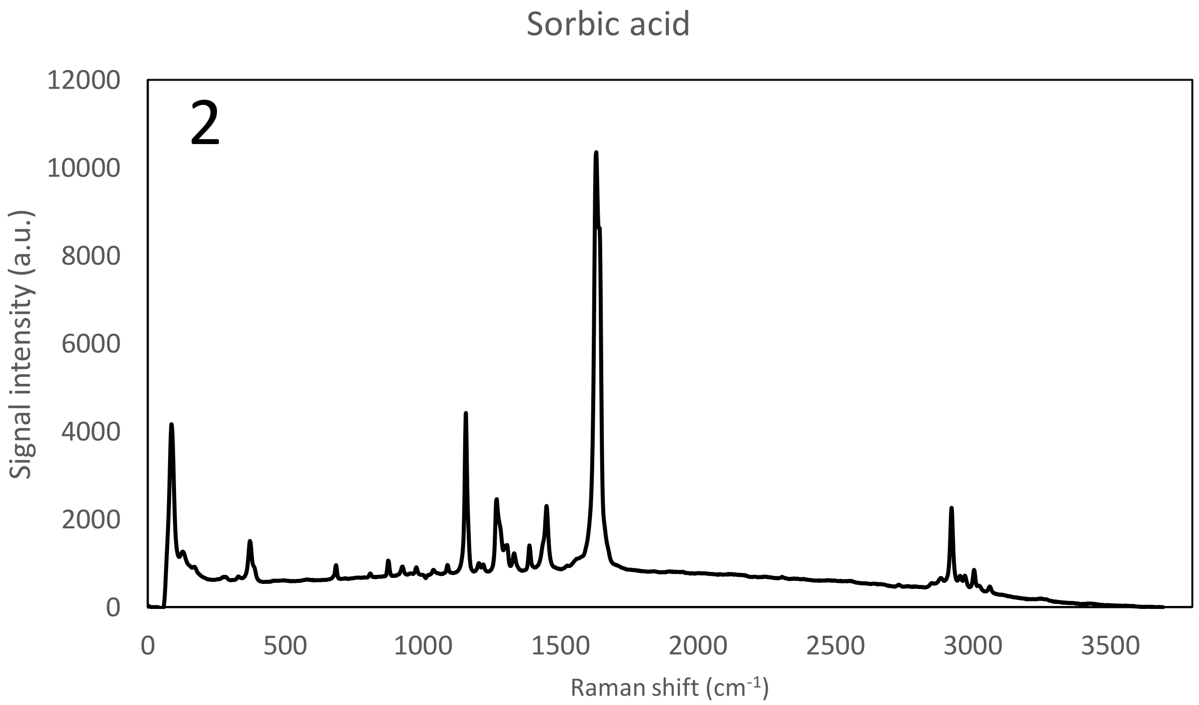

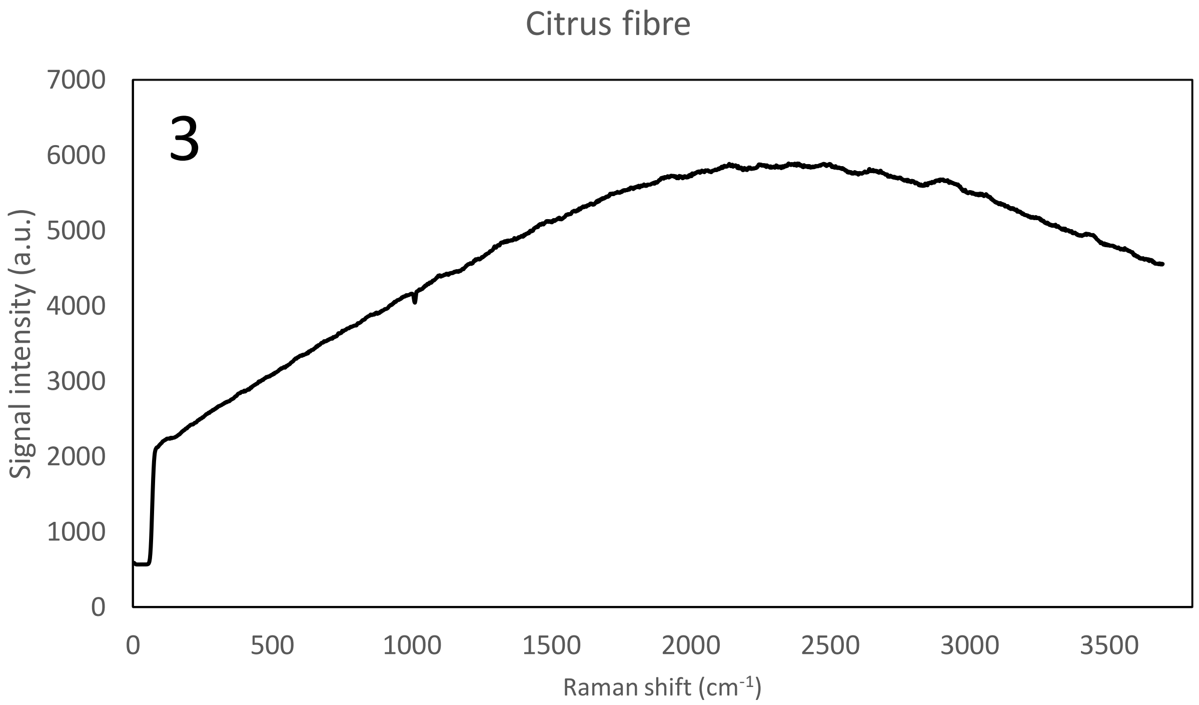
Citrus fibre
3
Signal intensity (a.u.)
Raman shift (cm-1)
0
1000
2000
3000
4000
5000
6000
7000
0
500
1000
1500
2000
2500
3000
3500

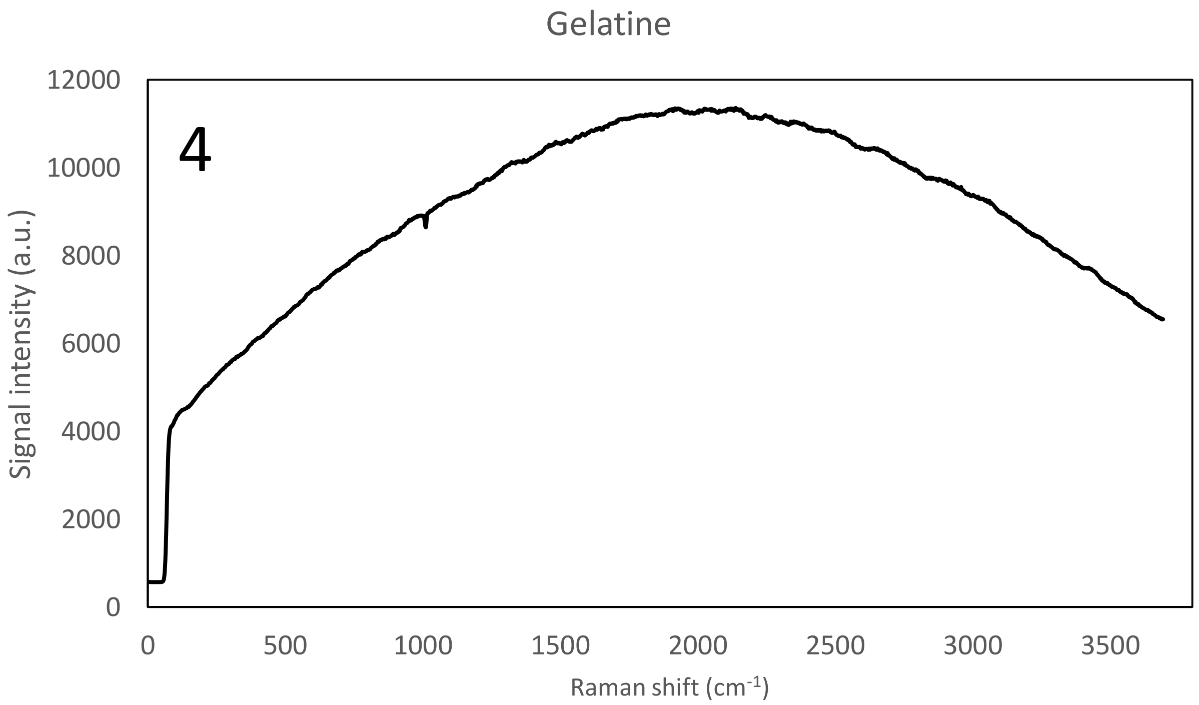
Gelatine
4
Signal intensity (a.u.)
Raman shift (cm-1)
0
2000
4000
6000
8000
10000
12000
0
500
1000
1500
2000
2500
3000
3500

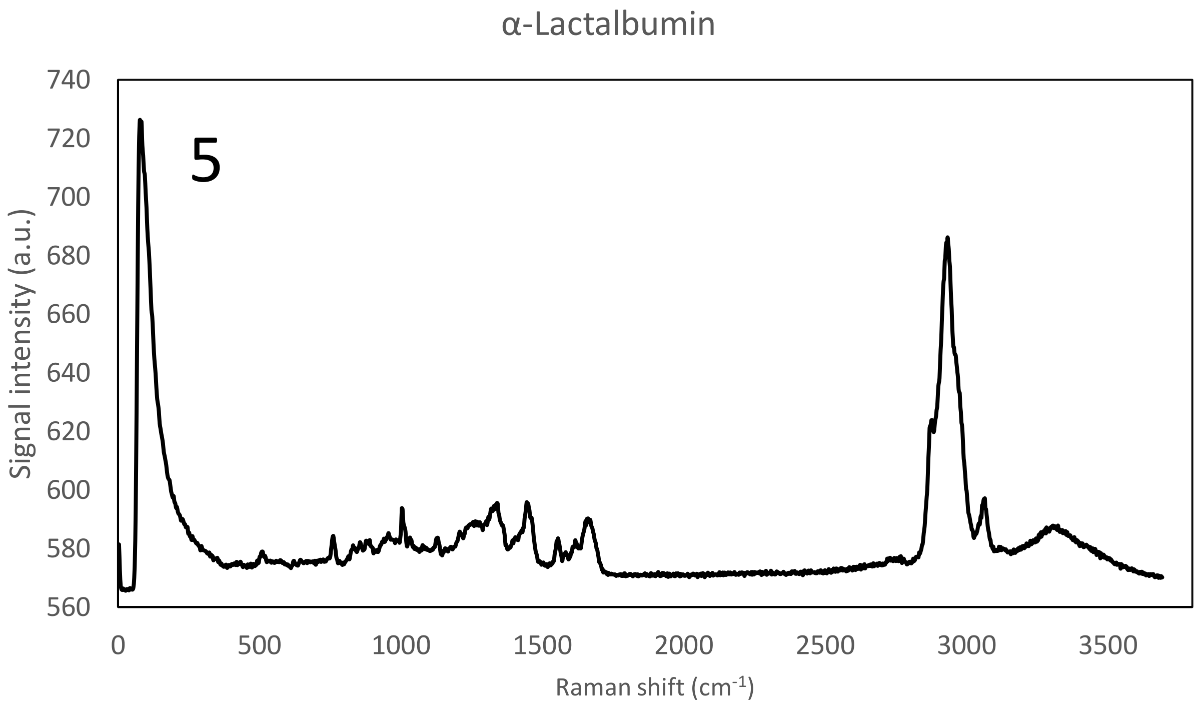

α-Lactalbumin
5
Signal intensity (a.u.)
740
720
700
680
660
640
620
600
580
560
0
500
1000
1500
2000
2500
3000
3500
Raman shift (cm-1)

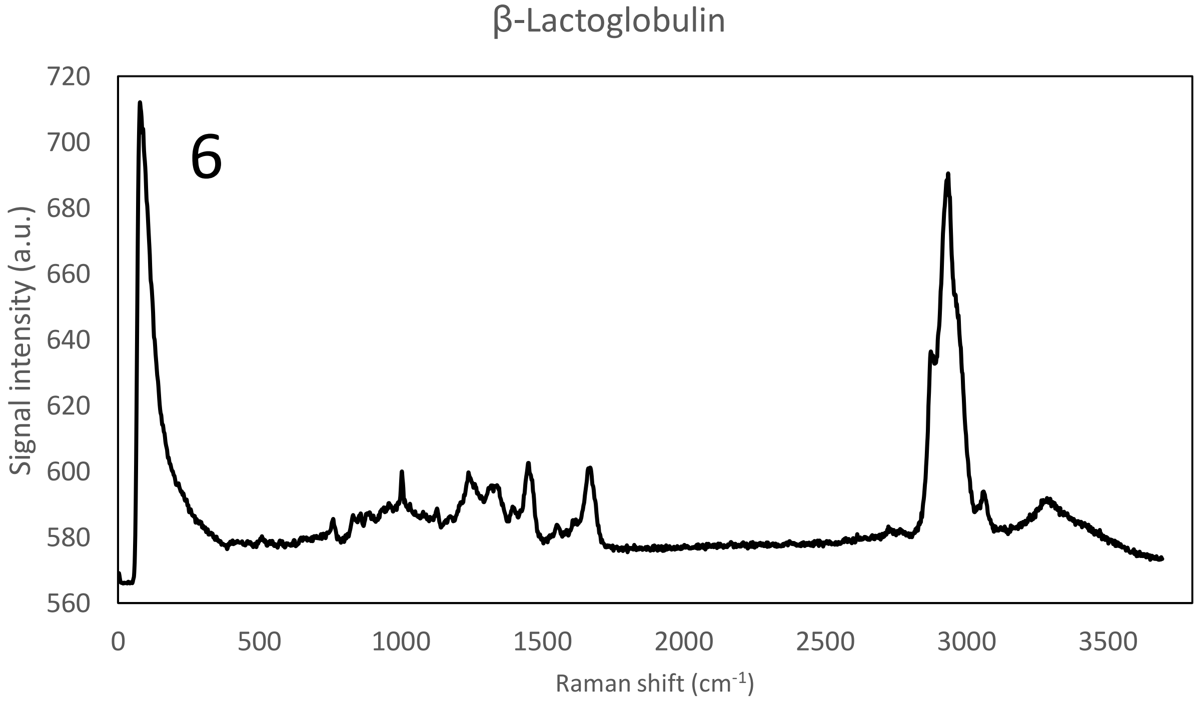

β-Lactoglobulin
6
Signal intensity (a.u.)
720
700
680
660
640
620
600
580
560
0
500
1000
1500
2000
2500
3000
3500
Raman shift (cm-1)

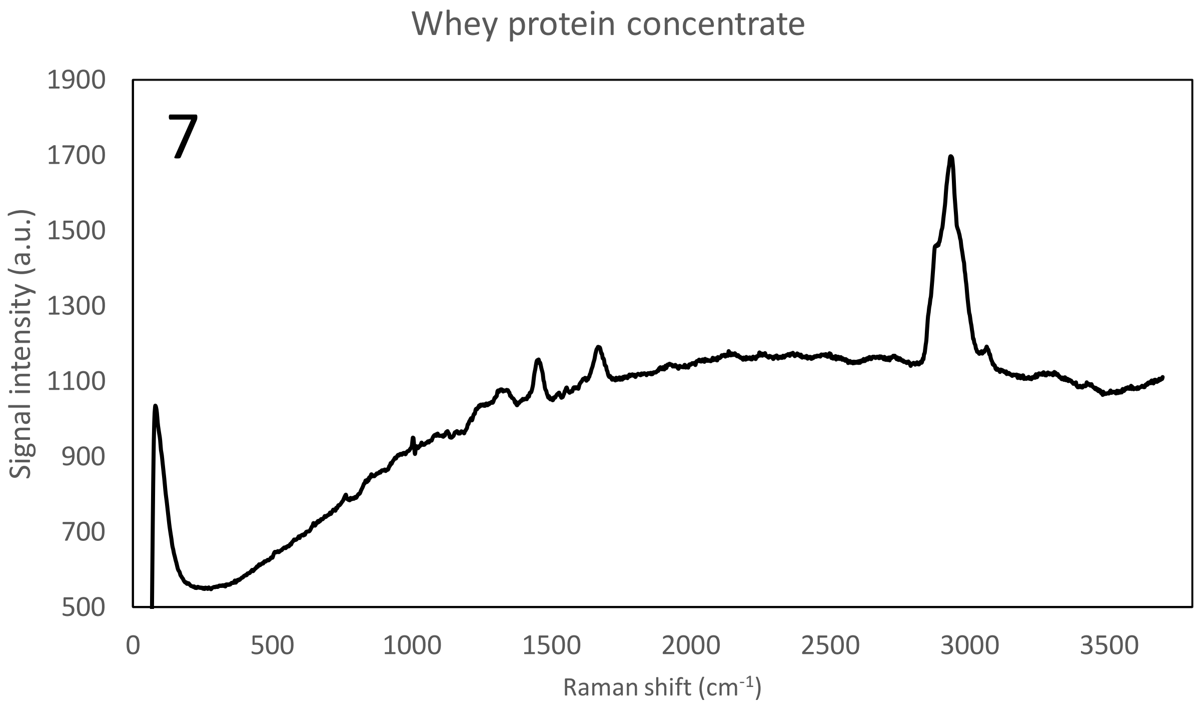
Whey protein concentrate
7
Signal intensity (a.u.)
1900
1700
1500
1300
1100
900
700
500
0
500
1000
1500
2000
2500
3000
3500
Raman shift (cm-1)

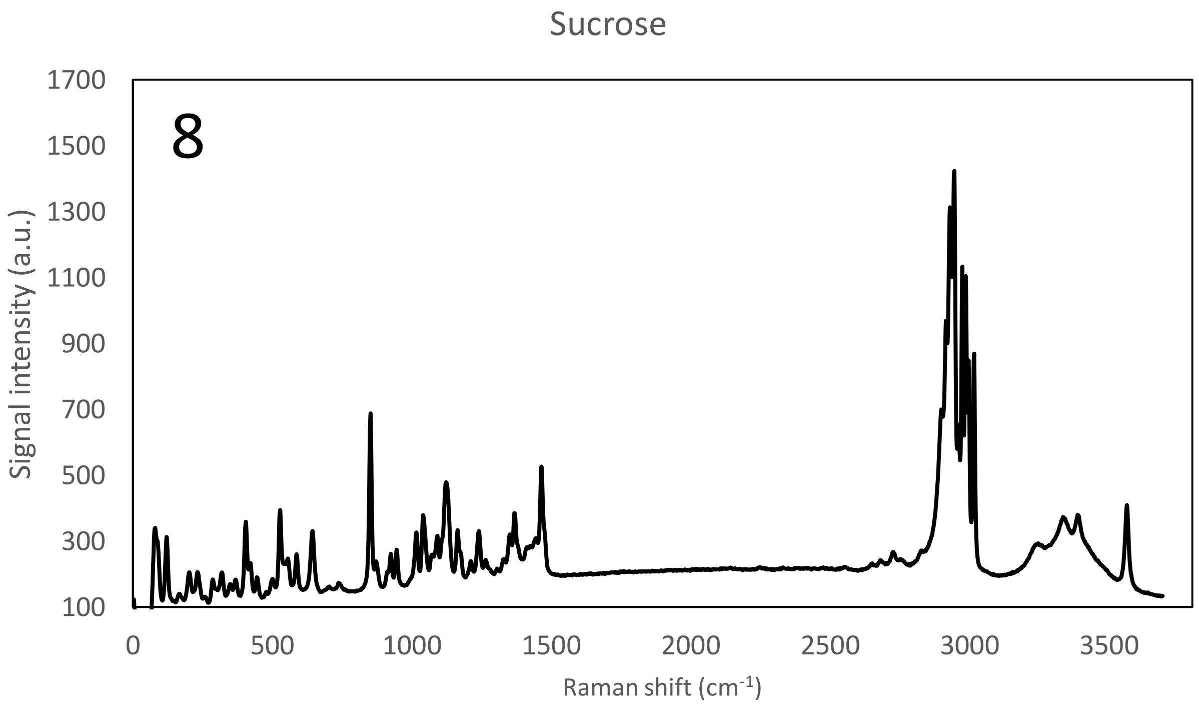
Sucrose
8
Signal intensity (a.u.)
1700
1500
1300
1100
900
700
500
300
100
0
500
1000
1500
2000
2500
3000
3500
Raman shift (cm-1)

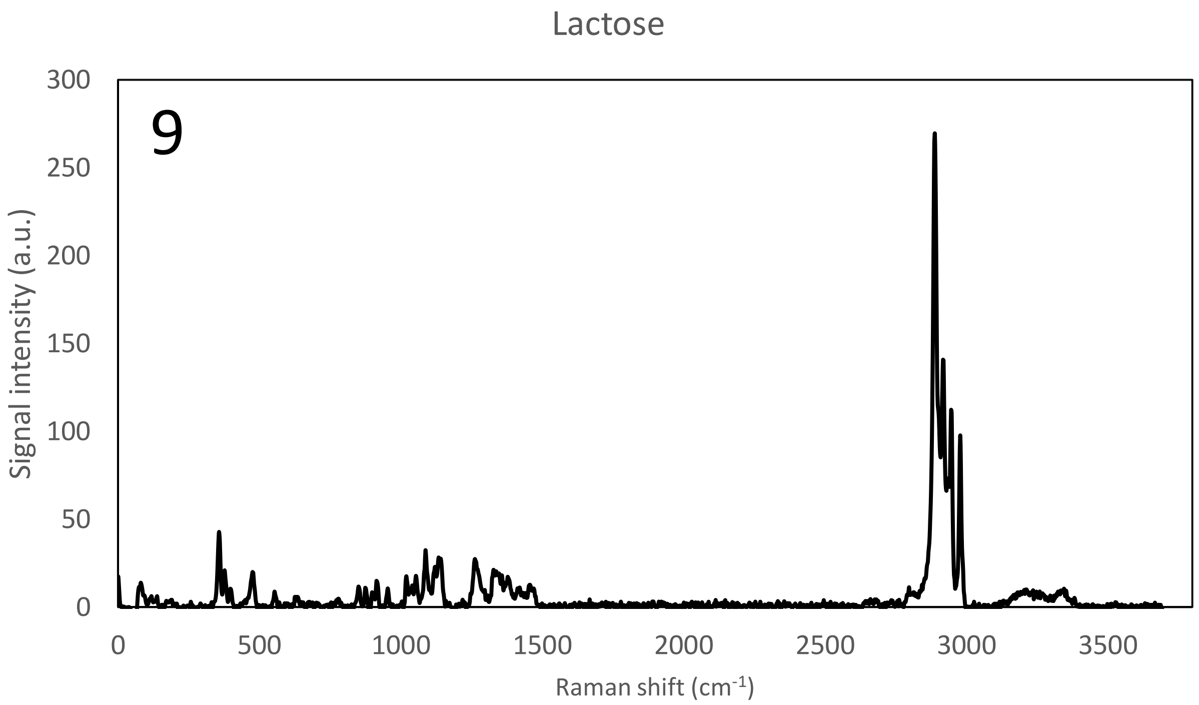
Lactose
9
300
250
200
150
100
50
0
Signal intensity (a.u.)
0
500
1000
1500
2000
2500
3000
3500
Raman shift (cm-1)

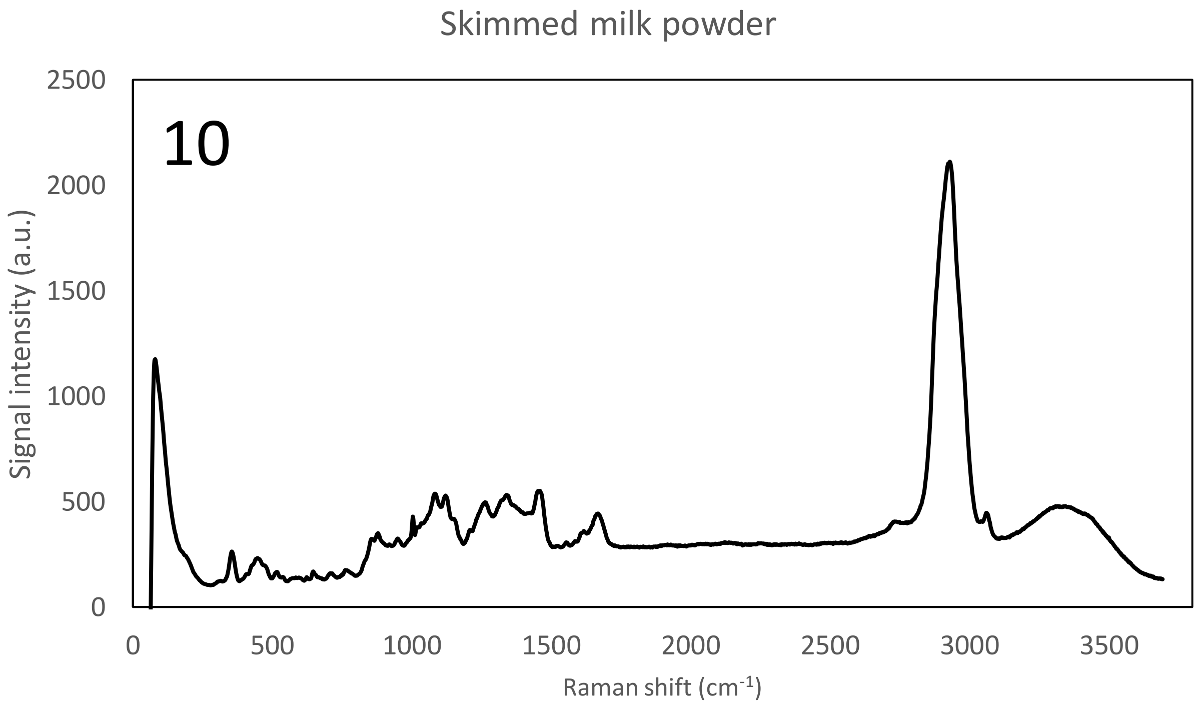
Skimmed milk powder
10
2500
2000
1500
1000
500
0
Signal intensity (a.u.)
0
500
1000
1500
2000
2500
3000
3500
Raman shift (cm-1)

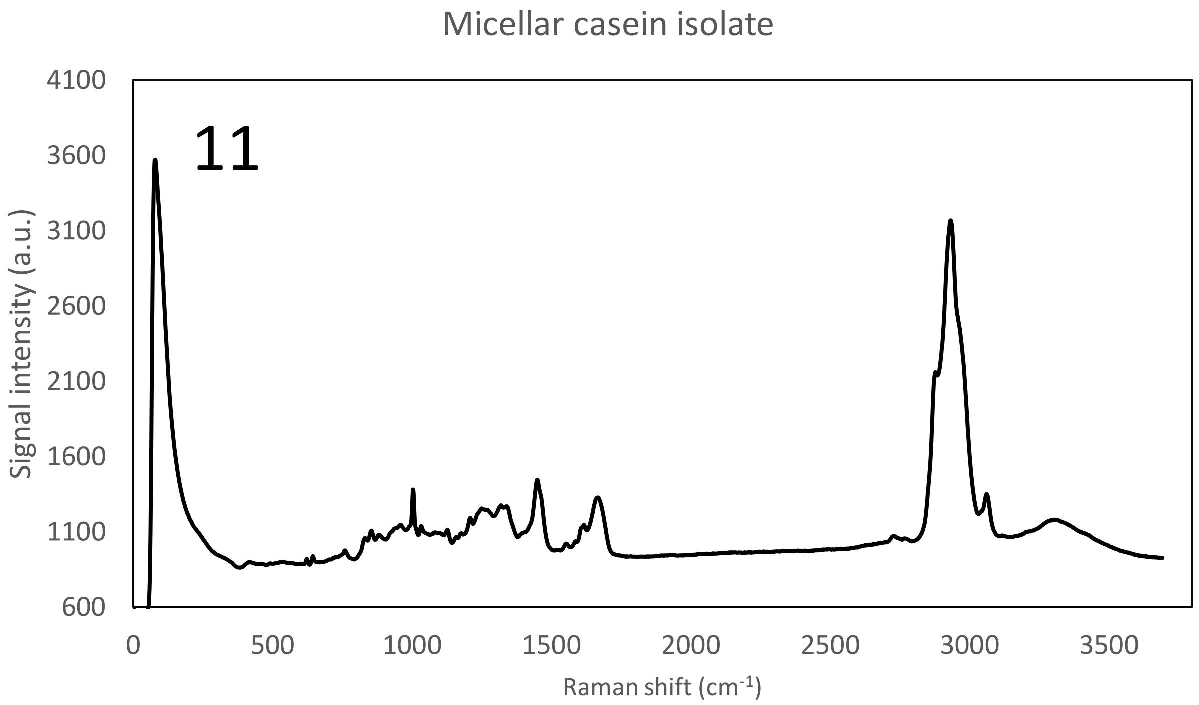

Micellar casein isolate
11
Signal intensity (a.u.)
600
1100
1600
2100
2600
3100
3600
4100
0
500
1000
1500
2000
2500
3000
3500
Raman shift (cm-1)

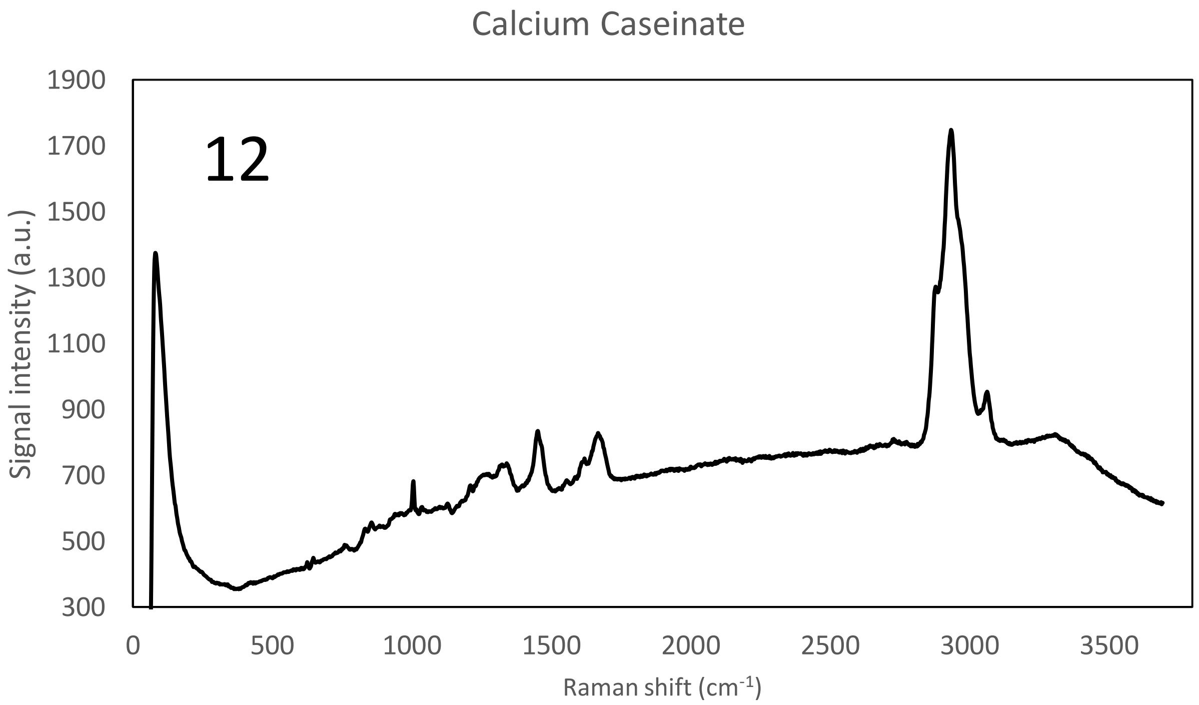

Calcium Caseinate
12
Signal intensity (a.u.)
300
500
700
900
1100
1300
1500
1700
1900
0
500
1000
1500
2000
2500
3000
3500
Raman shift (cm-1)

Figure S13-14. Raman fingerprint region (500-1800 cm^{-1}) of protein components: 13) Calcium caseinate, 14) Whey protein concentrate. A) Phenylalanine's benzene ring breathing (1000-5 cm-1); B) Amide III (1270 cm^{-1}); C) Tyrosine (1614 cm^{-1}); D) Amide I (1650-70 cm^{-1}).

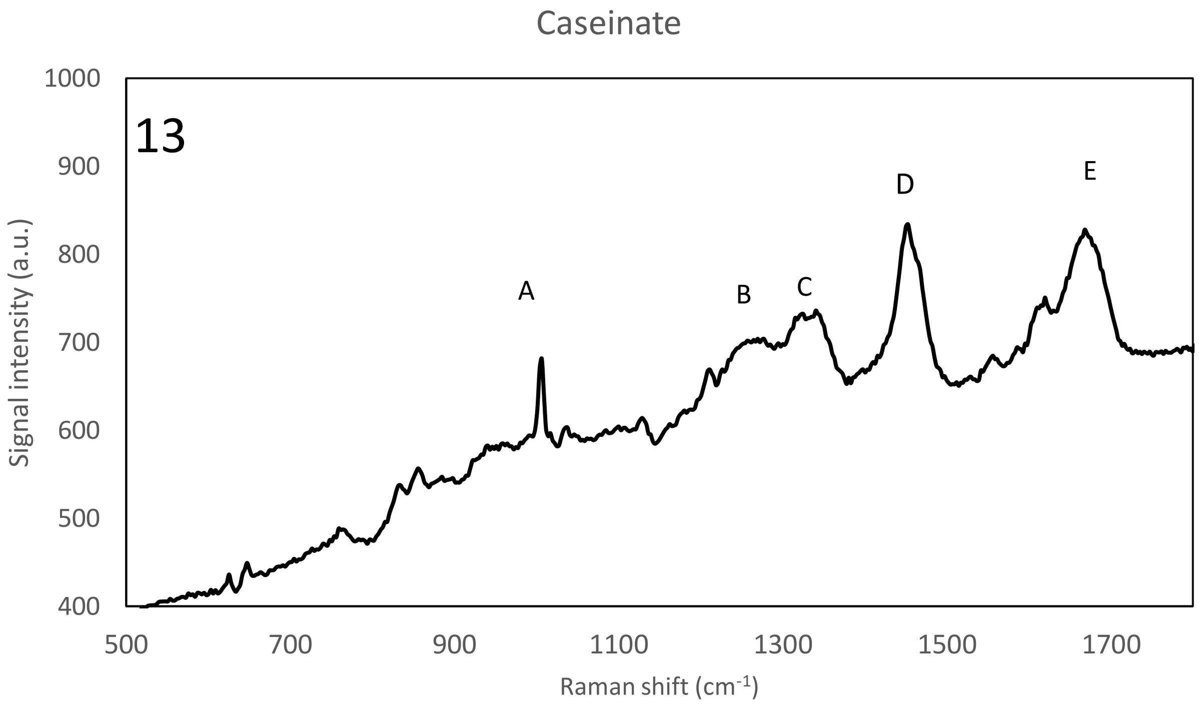

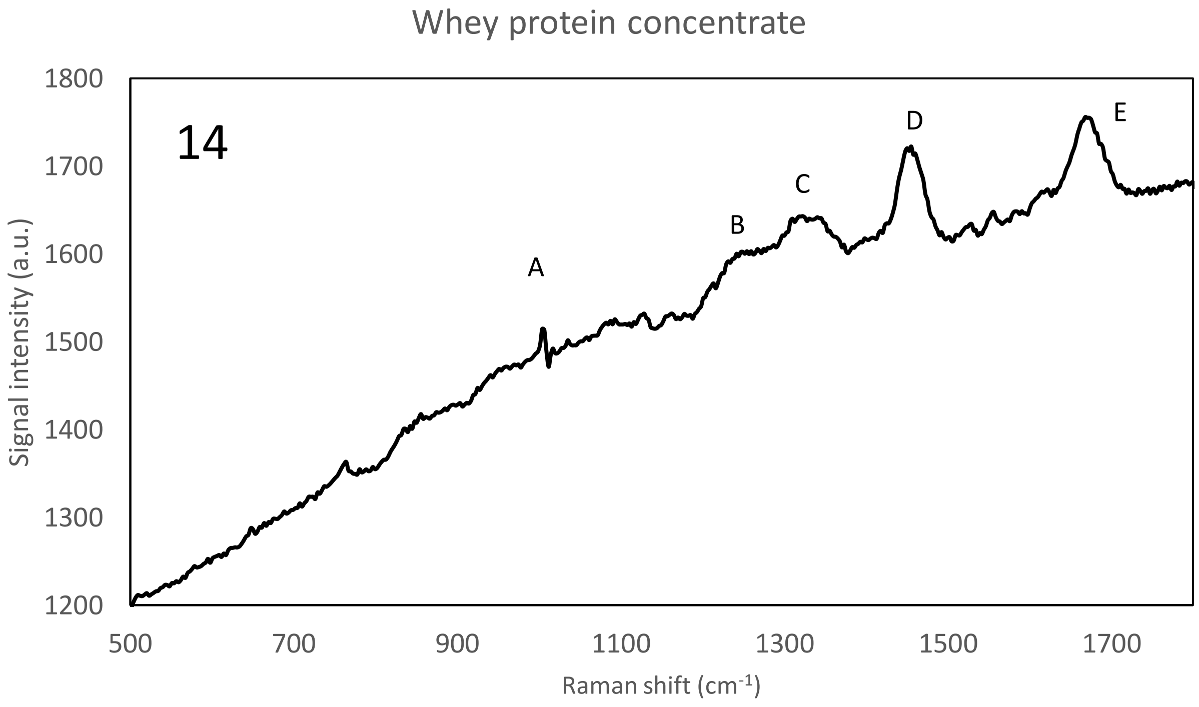

Figure S15. Supplementary Confocal Raman micrographs of cream cheeses. Cream cheese A before freezing (A), after freezing (B). Cream cheese B before freezing (C), after freezing (D). Red: fat. Green: protein. Blue: Water.

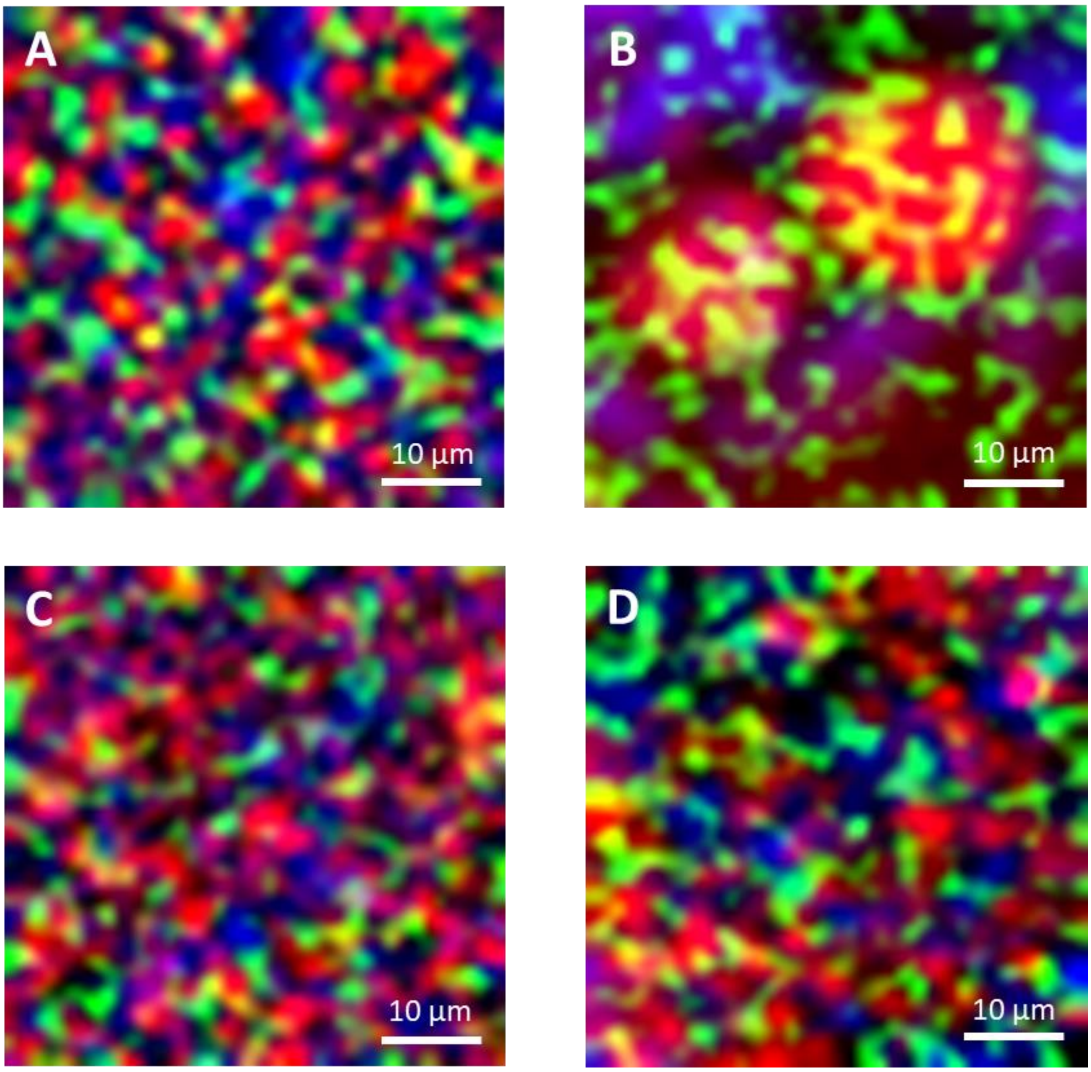

Figure S16. Supplementary Confocal laser scanning micrographs of cream cheeses. Cream cheese A before freezing (A), after freezing (B). Cream cheese B before freezing (C), after freezing (D). Red: fat. Green: protein.

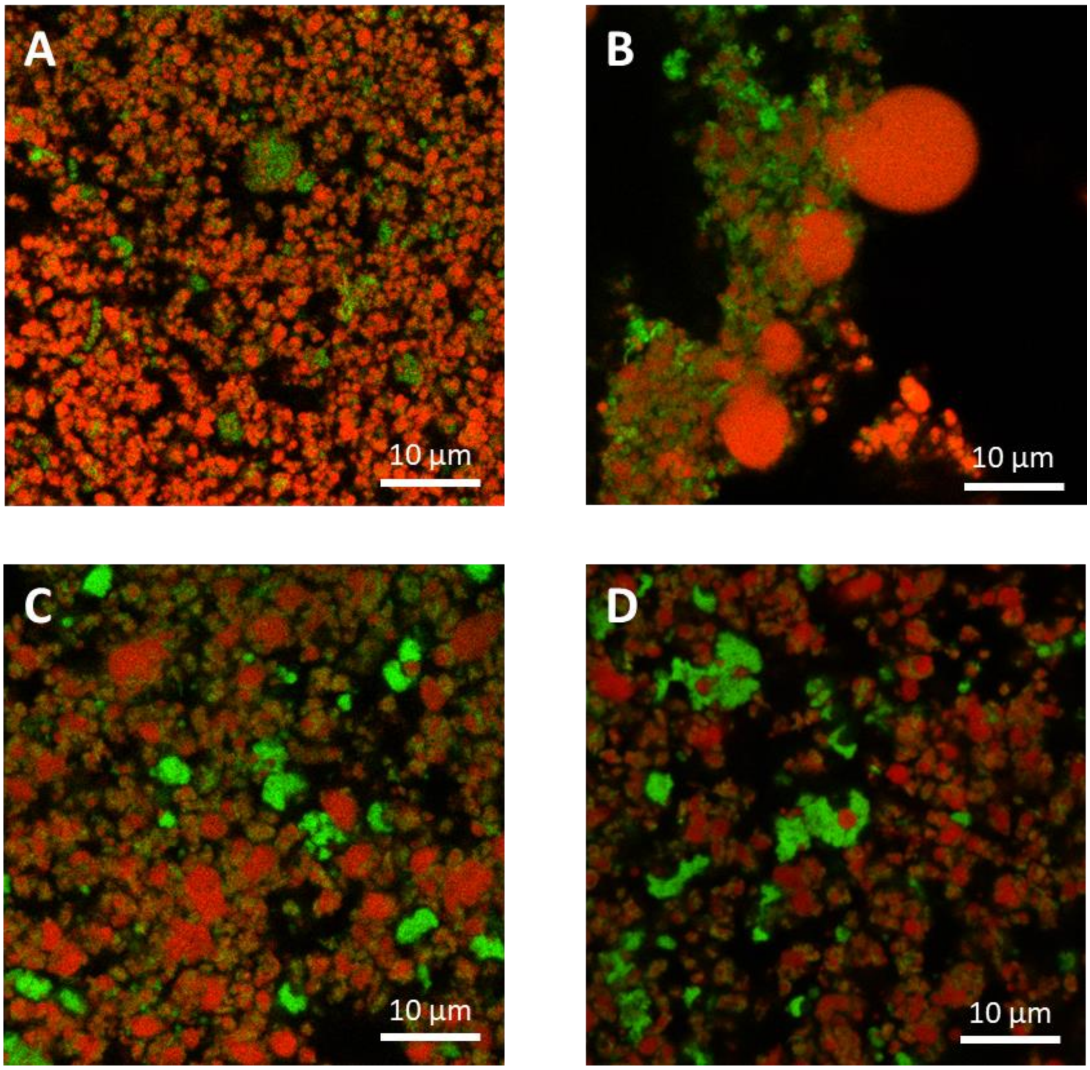

Supplement: Supplementary file 1 [file foods-09-00679-s001.pdf]
